# Factors associated with the improvement of the empathy levels among clinical-year medical students in Southern Thailand: a university-based cross-sectional study

**DOI:** 10.1186/s40359-022-00842-4

**Published:** 2022-05-21

**Authors:** Katti Sathaporn, Jarurin Pitanupong

**Affiliations:** Present Address: Department of Psychiatry, Faculty of Medicine, Prince of Songkla University, Hat Yai, 90110 Songkhla Thailand

**Keywords:** Burnout, Empathy, Factor, Mental health, Medical student

## Abstract

**Background:**

Empathy is one of the core medical professionalisms that distress, burnout, and lack of personal well-being is also recognized as an important influencer on lower empathy levels. Therefore, this study aimed to explore the mental health, burnout, and factors associated with the empathy levels among Thai, clinical-year medical students.

**Methods:**

This cross-sectional study surveyed all fourth-to sixth-year medical students at the Faculty of Medicine, Prince of Songkla University, in Thailand; at the end of the 2020 academic year. The questionnaires utilized were: (1) The personal and demographic information questionnaire, (2) The Toronto Empathy Questionnaire, (3) Thai Mental Health Indicator-15, and (4) The Maslach Burnout Inventory; Thai version. All data were analyzed using descriptive statistics, and factors associated with empathy levels were analyzed via the chi-square test and logistic regressions.

**Results:**

There were 466 participants, with a response rate of 91.5%. The majority were female (56.2%), and reported a below-average level of empathy (57.1%); with a median score (IQR) of 44 (40–48). The gender proportion of a below-average empathy level among male and female participants was 66.3% and 50.4%, respectively. Of the participants, 29.6% had poor mental health, 63.5% and 39.7% reported a high level of emotional exhaustion and depersonalization scores; even though most of them (96.6%) perceived having a high level of personal accomplishment. Multivariate analysis indicated that females, higher mental health, and a low level of depersonalization were statistically significant protective factors, which improved the empathy levels.

**Conclusions:**

More than half of the clinical-year medical students reported below-average empathy levels. Female gender, better mental health, and a low level of depersonalization were related to the improvement of the empathy levels. Therefore, medical educators should emphasize the importance of focusing supporting students, of all genders and in regards to all stages of medical education, to increase their levels of empathy, to promote individual well-being, and to effectively prevent the phenomenon of student ‘burnout’.

**Supplementary Information:**

The online version contains supplementary material available at 10.1186/s40359-022-00842-4.

## Background

Throughout medical education, empathy is one of the core professionalisms. It is the capacity to place oneself in another's position [1] and to feel or understand what another person is experiencing from within their frame of reference [2] that facilitates the understanding of the emotions of another person [3, 4]. Therefore, empathy is an emotional experience between an observer and a subject; in which, the observer, based on visual and auditory cues, identifies and transiently experiences the subject’s emotional state [5, 6]. In addition, the observer must convey that they not only identify with but also understand the basis of the subject’s feelings [7, 8]. This is because most parts of successful treatment depend on effective patient-physician interactions. Physicians who understand their patients on a personal level stand a better chance of experiencing and conveying empathy, as well as treating said patient more effectively, than a physician who does not have this level of understanding [9].

Even though empathy is a major component of a satisfactory physician–patient relationship, prior studies have suggested that medical students’ empathy levels might decline throughout clinical training. The main themes influencing the development and expression of empathy, within the medical education curriculum, include a course or subject; detailing the student’s character, patient’s profile, and surrounding conditions [10, 11]. Due to the nature of courses and approaches within medical education; such as hands-on experience, role models, science and theory [10], clinical training, and emphasis on the importance of empathy, these can negatively impact empathy [11]. The hypothesis might be that the training curriculum itself contributes to a decline in compassion after starting medical school [10, 11]. Additionally, surrounding conditions; time pressures, stress, job dissatisfaction, professional distance, work environments, and a hidden curriculum of cynicism may also contribute to these problems [12–14]. In concerns to the student’s character; maturity, personal level of empathy, and insecurities were factors associated with the impact of compassion for training [10, 12]. Moreover, perception of being abused or mistreated [15], distress, which included depression, anxiety, and lack of personal well-being, have also been recognized as exerting an important influence on practice habits [16, 17], performance, or lower levels of empathy among medical students [18].

Concerning the factors affecting empathy with patients among medical students, the prior study identified “perspective-taking”, “compassionate care” and “standing in patient's shoes” programs providing medical students to familiarize them with the importance of empathy and its role in care [19]. However, organizational culture, personal and interpersonal, and demographic factors are strongly implicated in inhibiting empathy [20]. Moreover, burnout, a syndrome characterized by three dimensions: feelings of energy depletion or exhaustion; increased mental distance from one’s job, or feelings of negativism or cynicism related to one's job as well as reduced professional efficacy [21], is one factor that influences the demonstration of empathetic behavior of physicians toward patients [20, 22]. Additionally, gender, values, emotions and coping strategies, quality of life [14], increased workload [13], patient behavior, inappropriate role modeling, informal or experiential learning, and lack of organizational support are also related to empathy level [22]. However, empathy may be a protective factor against burnout in physicians. Because empathy also positively impacts the physicians' quality of life, mental health and well-being: physicians evaluate an empathic relationship with patients as generating greater professional satisfaction [23].

In Thailand, one study was conducted in 2012, that identified the levels of empathy among medical students, and illustrated those that were female and preclinical-level students had higher empathy scores than their male and clinical-level counterparts. Notwithstanding this, that study did not identify any factors that correlate with empathy levels [23]. However, a recent study surveying preclinical-level and clinical-level medical students in 2021, identified preclinical-level students as having a higher level of empathy than clinical-level students [24]. Additionally, a study, conducted in three faculties of medicines in Thailand in the 2020 academic year, showed about 61% of sixth-year medical students had below-average empathy levels.

As mentioned above, recent studies in Thailand have suggested that in regards to medical students, empathy might decline due to their clinical training; it could also be suggested that there is a ‘hidden curriculum of cynicism’, which contributes to a decline of empathy levels. If this is true then it begs the question of why and how. Therefore, our study aimed to determine whether lower levels of empathy among clinical-level medical students are associated with mental health, burnout, and clinical-year level.

Findings could potentially provide useful information that can support efforts to enhance empathy, promote well-being, reduce distress among medical students, and establish an educational program about medical professionalism for each academic level.

## Methods

After approval from the corresponding Human Research Ethics Committee of the Faculty of Medicine, Prince of Songkla University (REC: 64-135-3-1), this cross-sectional study was conducted on all fourth-to sixth-year medical students, studying at the Faculty of Medicine, Prince of Songkla University, Hat Yai Hospital Medical Education Center, and Yala Hospital Medical Education Center, in the six-year graduate medical programs; at the end of the 2020 academic year. There were a total of 509 medical students: 174 from the fourth-year, 181 from the fifth-year, and 154 from the sixth-year. To be included, participants had to meet the criteria of being a medical student, aged more than 20 years, and completing all of the questionnaires. Meanwhile, those who were foreign students (two Cambodian medical students), inconvenienced to participate, or decided to withdraw from the study were excluded.

The data were collected following relevant guidelines, via a paper-based process. The research assistant approached all medical students in class and handed them an information sheet; which delineated the rationale for the study and allotted time to complete the survey. They had at least 10–15 min to consider whether to collaborate in the study or not. If they wished to participate, the research assistant handed them the questionnaires. Adhering to the policy of strict confidentiality, the signatures of the participants were not required, and they retained the right to withdraw from the research at any time without having to provide any explanation. All participants were allowed to finish, and return the questionnaires immediately or at a later time. They could submit the questionnaires via two options: drop them in the case at the front of the classroom, or return and place them in the case located at the Psychiatry Department. Therefore, protecting respondent confidentiality was retained. Additionally, the data were stored in a secure place, and only the researcher could access them via a password.

### Measures

(1) The personal and demographic information questionnaire consisted of questions related to age, gender, religion, income, cumulative GPA, history of alcohol consumption and/or substance use, physical and psychiatric illness, having experienced stress within the last year, and specialty preference.

(2) The Toronto Empathy Questionnaire (TEQ), which was translated to Thai, and was used to evaluate empathy, consisted of 16 questions and employed a 5-point rating scale for each question. The item responses were scored according to the following scale for positively worded items: 0 (never); 1 (rarely); 2 (sometimes); 3 (often); and 4 (always). The same scale was applied to reverse score negatively worded items. The scores of all 16 questions were summed, and they ranged from 0 to 64. Higher scores indicated higher levels of self-reported empathy, while total scores, below 45, were indicative of below-average empathy levels. The Cronbach’s alpha coefficient for this tool was 0.85. Empathy was divided into six subgroups: perception of an emotional state in another that stimulates the same emotion in oneself; assessment of emotion comprehension in others; assessment of emotional states in others by indexing the frequency of behaviors demonstrating appropriate sensitivity; sympathetic physiological arousal; altruism; behaviors engaging higher-order empathic responding; such as pro-social helping behavior [25].

(3) Thai Mental Health Indicator-15 (TMHI-15) consisted of 15 questions. The score of each question ranged from 1 to 4, and the total score was between 15 to 60. The interpretation of the total score was as follows: less than 43 (poor mental health), 44–50 (fair mental health), 51–60 (good mental health). This tool had a Cronbach’s alpha coefficient of 0.7 [26].

(4) The Thai version of the Maslach Burnout Inventory (MBI) questionnaire [27, 28]. This consisted of 22 items, divided into three dimensions: emotional exhaustion (feelings of being emotionally overextended and exhausted by one’s work), depersonalization (unsympathetic and impersonal responses toward the recipients of one’s care or service), and personal accomplishment (feelings of competence and achievement in one’s work with people) [28]. For the emotional exhaustion and depersonalization subscales, higher mean scores corresponded to higher degrees of burnout (emotional exhaustion score: 0–16 = low, 17–26 = moderate, > 26 = high; depersonalization score: 0–6 = low, 7–12 = moderate, > 12 = high). Lower mean scores of personal accomplishment corresponded to higher degrees of burnout (personal accomplishment score: > 38 = low, 32–38 = moderate, 0–31 = high). The Cronbach’s alpha coefficient of each domain, in the Thai version of MBI, was between 0.65–0.92 [27–29].

### Statistical methods

Descriptive statistics; such as proportions, means, standard deviation (SD), median and interquartile range (IQR) were calculated. Chi-square or Fisher’s exact tests and logistic regression analyses were used to identify associations between demographic characteristics, mental health, and burnout with the level of empathy. The correlation between each pair of explanatory variables was explored accordingly. If two variables were highly correlated, then this could indicate a possible source for multicollinearity. Furthermore, we considered the removal of a number of highly correlated independent variables. The analyses were conducted using R version 3.4.1 (R Foundation for Statistical Computing). Statistical significance was defined as a p-value of less than 0.05.

## Results

### Demographic characteristics

The fourth-to sixth-year medical students who completed the questionnaires comprised 466 of the 509 total medical students that were approached; the response rate was 91.6%. Of these, 152 (32.6%), 160 (34.3%), and 154 (33.0%) were fourth, fifth, and sixth-year, respectively. Demographic characteristics were shown in Table 1. The majority of participants were female (56.2%), and Buddhist (78.5%). Overall, their mean age was 23.1 ± 1.4 years, and the accumulative GPA was 3.2 ± 0.3. No statistically significant difference in demographic data (gender, religion) was observed between the medical students, according to clinical-year level.

**Table 1:** Demographic characteristics, mental health, and burnout categorized by the level of empathy

Variables	Total (n = 466)	Level of empathy		Chi^2^ P-value
		< 45	≥ 45	
		(n = 266)	(n = 200)	
*Gender*				< 0.001
Male	202 (43.3)	134 (50.4)	68 (34.3)	
Female	262 (56.2)	132 (49.6)	130 (65.7)	
No answer	2 (0.4)			
*Religion*				0.521
Buddhism	366 (78.5)	211 (86.5)	155 (83.8)	
Islam, Christianity, others	63 (13.5)	33 (13.5)	30 (16.2)	
No answer	37 (7.9)			
*Physical illness*				0.994
No	396 (85.0)	226 (85.0)	170 (85.4)	
Yes	69 (14.8)	40 (15.0)	29 (14.6)	
No answer	1 (0.2)			
*Psychiatric illness*				0.371
No	420 (90.1)	241 (92.3)	179 (89.5)	
Yes	41 (8.8)	20 (7.7)	21 (10.5)	
No answer	5 (1.1)			
*Alcohol consumption*				0.191
No	311 (66.7)	184 (69.7)	127 (63.5)	
Yes	153 (32.8)	80 (30.3)	73 (36.5)	
No answer	2 (0.4)			
*Substance use*				1^a^
No	459 (98.5)	262 (99.2)	197 (99.5)	
Yes	3 (0.6)	2 (0.8)	1 (0.5)	
No answer	4 (0.9)			
*Specialty preference*				0.195
General/not specified	158 (33.9)	98 (36.8)	60 (30.0)	
Major	200 (42.9)	113 (42.5)	87 (43.5)	
Minor	108 (23.2)	55 (20.7)	53 (26.5)	
*Clinical year*				0.039
4^th^ Year	152 (32.6)	75 (28.2)	77 (38.5)	
5^th^ Year	160 (34.3)	93 (35.0)	67 (33.5)	
6^th^ Year	154 (33.0)	98 (36.8)	56 (28.0)	
*Mental health*				< 0.001
Poor	138 (29.6)	103 (38.7)	35 (17.5)	
Fair	237 (50.9)	140 (52.6)	97 (48.5)	
Good	91 (19.5)	23 (8.6)	68 (34.0)	
*Burnout*				
Emotional exhaustion				0.043
Low	52 (11.2)	23 (8.7)	29 (14.5)	
Moderate	115 (24.7)	60 (22.8)	55 (27.5)	
High	296 (63.5)	180 (68.4)	116 (58.0)	
No answer	3 (0.6)			
Depersonalization				< 0.001
Low	143 (30.7)	59 (22.4)	84 (42.0)	
Moderate	135 (29.0)	83 (31.6)	52 (26.0)	
High	185 (39.7)	121 (46.0)	64 (32.0)	
No answer	3 (0.6)			
Personal accomplishment				0.077
Low	–	–	–	
Moderate	13 (2.8)	11 (4.2)	2 (1.0)	
High	450 (96.6)	252 (95.8)	198 (99.0)	
No answer	3 (0.6)			
*Having stress within 1 year*				0.253
No	21 (4.5)	15 (5.7)	6 (3.0)	
Yes	444 (95.5)	250 (94.3)	194 (97.0)	
No answer	1 (0.2)			

^a^Fisher's exact test

### Empathy level

**Fig. 1:**
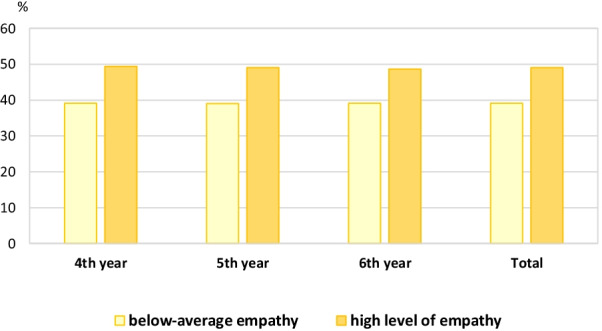
Empathy level according to clinical-year level (n = 466). Note: Statistically significant difference between clinical-year, p-value = 0.039. Definition: below-average empathy = empathy score < 45, high level of empathy = empathy score  ≥ 45

The Toronto Empathy Questionnaire results revealed that 266 (57.1%) participants reported a below-average empathy level (Table 1 and Fig. 1). The median TEQ score (IQR) was 44 (40–48). Concerning a below-average empathy level, the gender proportion among male and female participants was 66.3% and 50.4%, respectively. Regarding the clinical-year level, the median TEQ score (IQR) of fourth-to sixth-year was 45 (40–48.2), 43 (40–48), 43 (39–46), respectively (Table 2). A statistically significant difference in the total scores of empathy level was observed between the students according to clinical-year level via univariate analysis (P-value = 0.039) (Fig. 1).

**Table 2:** Subgroups of empathy according to the Toronto Empathy Questionnaire

Domain of empathy	Median (IQR)			
	Total	4th year	5th year	6th year
	(n = 466)	(n = 152)	(n = 160)	(n = 154)
Perception of an emotional state in another that stimulates the same emotion in oneself	2.5 (2.0–3.0)	2.5 (2.0–3.0)	2.5 (2.5–3.0)	2.5 (2.0–3.0)
Assessment of emotion comprehension in others	3.0 (2.0–3.0)	3.0 (2.0–3.0)	3.0 (2.0–3.0)	3.0 (2.0–3.0)
Assessment of emotional states in others by indexing the frequency of behaviors demonstrating appropriate sensitivity	2.6 (2.4–3.0)	2.8 (2.4–3.2)	3.0 (2.6–3.2)	2.6 (2.4–3.0)
Sympathetic physiological arousal*	2.8 (2.5–3.0)	2.7 (2.5–3.0)	2.8 (2.5–3.2)	2.8 (2.5–3.0)
Altruism	3.0 (2.7–3.3)	3.0 (2.7–3.7)	3.3 (3.0–3.7)	3.0 (2.7–3.3)
Behaviors engaging higher-order empathic responding*	2.0 (2.0–3.0)	3.0 (2.0–3.0)	3.0 (2.0–3.0)	2.0 (2.0–3.0)
Total score *	44.0 (40.0–48.0)	45.0 (40.0–48.2)	43.0 (40.0–48.0)	43.0 (39.0–46.0)

*Statistically significant difference between clinical-year (Kruskal–Wallis test p-value < 0.05

According to six TEQ subgroups, the results showed that the assessment of emotion comprehension in others and altruism had the highest median TEQ subgroup score (IQR) [3 (2–3) and 3 (2.7–3.3), respectively]; whereas, behaviors engaging higher-order empathic responding exhibited the lowest median TEQ subgroup score (IQR) [2 (2–3)]: especially in sixth-year participants. A statistically significant difference in the level of empathy; sympathetic physiological arousal and behaviors engaging higher-order empathic responding were detected between the medical students according to the clinical-year level (p-value < 0.05) (Table 2).

### Mental health

According to the Thai Mental Health Indicator-15, 237 (50.9%) participants had fair mental health, and 138 (29.6%) participants had poor mental health (Table 1). No statistically significant difference in mental health was detected between the medical students according to the clinical-year level (Table 3). Out of 41 participants who had psychiatric illnesses, 19 and 22 participants had poor and fair mental health, respectively. Concerning perceived stress, 444 (95.5%) participants reported having stress within a previous year. The most common stresses were medical course and examination (92.8%), learning environments (45.7%), and relationships with friends (29.1%).

**Table 3:** Demographic characteristics, mental health, burnout and empathy; categorized by clinical-year level

Demographic characteristics, mental health, burnout and empathy	Total (n = 466)	Number (%)			Chi^2^ P-value
		4th year	5th year	6th year	
		(n = 152)	(n = 160)	(n = 154)	
*Gender*					0.087
Male	202 (43.3)	70 (46.4)	76 (47.8)	56 (36.4)	
Female	262 (56.2)	81 (53.6)	83 (52.2)	98 (63.6)	
No answer	2 (0.4)				
*Religion*					0.775
Buddhism	366 (78.5)	120 (86.3)	129 (86.0)	117 (83.6)	
Islam, Christianity, others	63 (13.5)	19 (13.7)	21 (14.0)	23 (16.4)	
No answer	37 (7.9)				
*Physical illness*					0.010
No	396 (85.0)	118 (78.1)	144 (90.0)	134 (87.0)	
Yes	69 (14.8)	33 (21.9)	16 (10.0)	20 (13.0)	
No answer	1 (0.2)				
*Psychiatric illness*					0.580
No	420 (90.1)	135 (89.4)	143 (91.1)	142 (92.8)	
Yes	41 (8.8)	16 (10.6)	14 (8.9)	11 (7.2)	
No answer	5 (1.1)				
*Alcohol consumption*					0.113
No	311 (66.7)	101 (66.9)	98 (61.6)	112 (72.7)	
Yes	153 (32.8)	50 (33.1)	61 (38.4)	42 (27.3)	
No answer	2 (0.4)				
*Substance use*					0.777^a^
No	459 (98.5)	148 (100)	158 (98.8)	153 (99.4)	
Yes	3 (0.6)	0 (0)	2 (1.2)	1 (0.6)	
No answer	4 (0.9)				
*Specialty preference*					0.363
General / not specified	158 (33.9)	55 (36.2)	60 (37.5)	43 (27.9)	
Major	200 (42.9)	60 (39.5)	66 (41.2)	74 (48.1)	
Minor	108 (23.2)	37 (24.3)	34 (21.2)	37 (24.0)	
*Level of empathy*					0.039
< 45	266 (57.1)	75 (49.3)	93 (58.1)	98 (63.6)	
>45	200 (42.9)	77 (50.7)	67 (41.9)	56 (36.4)	
*Mental health*					0.209
Poor	138 (29.6)	38 (25.0)	54 (33.8)	46 (29.9)	
Fair	237 (50.9)	77 (50.7)	76 (47.5)	84 (54.5)	
Good	91 (19.5)	37 (24.3)	30 (18.8)	24 (15.6)	
*Burnout*					
Emotional exhaustion					0.092
Low	52 (11.2)	14 (9.3)	19 (11.9)	19 (12.4)	
Moderate	115 (24.7)	48 (31.8)	29 (18.2)	38 (24.8)	
High	296 (63.5)	89 (58.9)	111 (69.8)	96 (62.7)	
No answer	3 (0.6)				
Depersonalization					< 0.001
Low	143 (30.7)	59 (39.1)	50 (31.4)	34 (22.2)	
Moderate	135 (29.0)	50 (33.1)	49 (30.8)	36 (23.5)	
High	185 (39.7)	42 (27.8)	60 (37.7)	83 (54.2)	
No answer	3 (0.6)				
Personal accomplishment					0.094^a^
Low	–	–	–	–	
Moderate	13 (2.8)	8 (5.3)	2 (1.3)	3 (2.0)	
High	450 (96.6)	143 (94.7)	157 (98.7)	150 (98.0)	
No answer	3 (0.6)				
*Having stress within 1 year*					0.258
No	21 (4.5)	4 (2.6)	7 (4.4)	10 (6.5)	
Yes	444 (95.5)	148 (97.4)	153 (95.6)	143 (93.5)	
No answer	1 (0.2)				

^a^Fisher's exact test

### Burnout

**Fig. 2:**
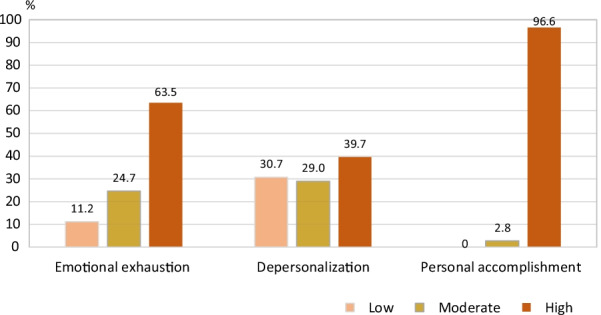
Burnout dimensions according to The Maslach Burnout Inventory (n = 466)

The Maslach Burnout Inventory-Thai version finding indicated that 296 (63.5%) had high emotional exhaustion, and another 185 (39.7%) had high depersonalization scores. No one perceived themselves as having low personal accomplishment, with almost all of them (96.6%) stating a high level of personal accomplishment (Table 1 and Fig. 2). The median score (IQR) for emotional exhaustion, depersonalization, and personal accomplishment were 31 (22–40), 10 (5–15), and 12 (8–17.7), respectively. According to the burnout subpart, a statistically significant difference in emotional exhaustion and depersonalization was detected between the medical students according to the empathy level (p < 0.05 and p-value < 0.001, respectively) (Table 1). Additionally, a statistically significant difference in depersonalization was also detected between the students according to clinical-year level (p < 0.001) (Table 3).

### Association between demographic characteristics, mental health, burnout, and level of empathy

To identify factors associated with the level of empathy, demographic characteristics, specialty preference, mental health, and burnout were included in the univariate analysis. Variables with p-values of less than 0.2 from the univariate analysis were included in the initial model of the multiple logistic regression analysis (Table 1).

The multivariate analysis indicated statistically significant factors that improved the level of empathy were; female, low depersonalization level, and having fair to good mental health. Females identified as having a higher empathy level than males, the adjusted odds ratio (AOR) was 1.92, and the 95% confidence interval (CI) was 1.27 to 2.91. Good mental health revealed a higher empathy level than poor mental health; the AOR (95%CI) was 7.93 (4.71,15.08). Low depersonalization showed a higher empathy level than high depersonalization; the AOR (95%CI) was 1.69 (1.03,2.77) (Table 4). The Variance Inflation Factor (VIF) indicated a value, which was below 5, and closer to 1, in regards to all variables.

**Table 4:** Factors associated with high levels of empathy

Factors	Crude OR (95% CI)	Adjusted OR (95% CI)	P-value LR-test
*Gender*			0.002
Male	Reference	Reference	
Female	1.96 (1.34,2.86)	1.92 (1.27,2.91)	
*Mental health*			< 0.001
Poor	Reference	Reference	
Fair	1.99 (1.25,3.17)	1.81 (1.12,2.93)	
Good	9.01 (4.87,16.67)	7.93 (4.71,15.08)	
*Depersonalization*			0.016
High	Reference	Reference	
Low	2.66 (1.69,4.17)	1.69 (1.03,2.77)	
Moderate	1.16 (0.73,1.84)	0.82 (0.49,1.36)	

## Discussion

This study indicated that more than half (57.1%) of fourth-to sixth-year medical students understudy had below-average empathy levels, as indicated by a median TEQ score (IQR) of 44 (40–48). Comparing the level of empathy discovered in our study with those reported by prior study, it was similar to the one found by a study in Malaysia [30]. However, our level of empathy was lower than those found by a study in Turkey [31]. These variances might be due to differences in population ethnicity, and culture. In this study, a statistically significant difference in the total score of empathy level was detected between medical students according to clinical-year level (P-value = 0.039) for univariate analysis. However, after adjusting for other variables in multivariate analysis, we didn’t found a statistically significant difference between groups. This might indicate that level of empathy was not reduced with clinical training, like those found by the prior studies [11, 30, 31]. Moreover, the study of empathy among medical students, conducted at a Korean medical school, also revealed that later years of training were associated with significantly better empathy [32].

In terms of empathy subgroups, empathy encompasses cognitive and affective or emotional dimensions. The cognitive dimension refers to ‘the ability to understand the patient’s inner experiences and perspectives, and a capability to communicate this understanding’ [33]; whereas, the affective dimension refers to ‘the ability to imagine the patient’s emotions and perspectives [34]. In this study, the assessment of emotion comprehension in others and altruism showed the highest empathy subgroup score; whereas, behaviors engaging higher-order empathic responding exhibited the lowest empathy subgroup score; especially in the sixth-year level. This might imply that most medical students were able to understand the patient’s inner experience and imagine their emotions or perspectives. However, they might lack the ability to express or transfer their empathy toward others.

It was agreed that effective expression of empathy or good communication skills on the part of physicians should enable them to convey their actual feelings or experiences to patients. Physicians who are poor communicators, and inept at expressing their feelings properly might be misunderstood by patients and people around them [35]. Then, the development and operation of empathy could be promoted by increasing: hands-on-experiences, possibilities to experience the patient’s point of view, and offering patient contact early in the curriculum. Besides these factors, students need support in reflecting on their actions, behavior, and experiences with patients. Additionally, instructors need time and opportunities to reflect on their communication with and treatment of patients, on their teaching behavior, and on their function as role models for treating patients empathically and preventing stress [10].

In this study, statistically significant differences in empathy levels were detected between male and female medical students. Females had statistically significant higher scores of empathy than males; this was similar to those reported by prior studies [30, 31, 36]. Regarding gender differences, educational intervention in all gender groups should be a cause for concern, because enhancing empathy levels will sequentially promote patient care [36]. All medical students should be educated in a way that they learn both scientific concepts of medicine, communicate with patients, and also learn how to empathize with them. They must learn how to treat patients, not just treat their diseases (37). Hence, medical school curriculums should go in the right direction, by focusing more on teaching adequate communication and interaction behaviors by covering all genders [10, 38].

As for mental health and burnout syndrome, this study identified the majority of clinical-year medical students revealed fair to good mental health, there was only 29.6% of them had poor mental health. The most common perceived stresses were medical course and examination, learning environments, and relationships with friends. Moreover, based on burnout syndrome, no one had a low personal accomplishment, and the majority of them perceived high personal accomplishment; even though they had high emotional exhaustion and high depersonaliszation scores. Additionally, statistically significant protective factors in regards to empathy levels were: female gender, good mental health, and low levels of depersonalization. Consequently, there were medical students with lower empathy levels without an association to all burnout dimensions. Medical students who had poor mental health and a high level of depersonalization, having high mental distance from one’s feelings, might have negative feelings and attitudes toward patients, together with negative school and/or work experiences. This may be one of the causes in regards to negative emotion expression or having low empathy levels [39].

Concerning the effect of gender in regards to the brain and human behavior and more specifically about empathy development; evidence suggests that there is male vs. female differences in connection to the capacity for empathy. Females are portrayed as being more nurturing and empathetic, while males are portrayed as being less emotional and more cognition based. These differences may affect how males and females’ respond in regards to the different roles that they may have [40]. According to these evidence, males may show higher levels of depersonalization or more mental distance from their feelings vs. females. This means that males may present as less empathic vs. females.

Finally, our medical education curriculum and educational environment should be reviewed, and the practical experiences should be made less stressful and promote good mental health for medical students [10]. Additionally, it should devote more time to empathy education to prevent the decrease in empathy levels, increase empathy [36], and develop the evidence-based guidelines on improving mental well-being in the workplace, prevention of depersonalization, or mental distance between medical students and patients in all genders and all educational phases.

This study had a few strengths and limitations worth mentioning. To our knowledge, this is the first study that explored mental health, and burnout, as potential associating factors with the level of empathy among Thai, clinical-year medical students. However, this study had some limitations. It was a cross-sectional survey and utilized self-administered questionnaires; therefore, some misunderstanding regarding the intended meaning of the questions might have occurred. Nevertheless, to minimize this, questionnaires with good reliability were utilized (good Cronbach’s alpha coefficient values). Regarding alcohol consumption and substance use, this study was limited by the aggregated data of substance use, because in Thailand all substances are illegal except kratom, THC, and CBD oil, alcohol drinking. Thus, we asked about history of substance abuse and alcohol drinking separately. Another drawback was that our data was quantitative, and the sample size was restricted to only medical students who graduated from one medical school. Hence, this dataset may not fairly represent the situation of all Thai medical students throughout the country.

Henceforward, studies are recommended to include all medical students attending all the faculties of medicine in Thailand. Therefore, a comprehensive multi-center study should be conducted. Moreover, other studies should employ more qualitative methods, survey medical students longitudinally, and include control groups.

## Conclusion

More than half of the clinical-year medical students had below-average empathy levels. However, they showed a higher score in the assessment of emotion comprehension in others and altruism; even though behaviors engaging higher-order empathic responding exhibited the lowest score. Female gender, better mental health, and low levels of depersonalization related to the improvement of the level of empathy. Therefore, medical educators should focus on measures that can increase empathy levels, for students of all genders and in all stages of their medical education, promoting well-being and preventing student ‘burnout’.

## Supplementary Information

**Additional file 1**. Raw data.

## Data Availability

All data generated or analysed during this study are included in this published article [and its Additional file 1].

## Abbreviations

MBI: Maslach Burnout Inventory
TEQ: The Toronto Empathy Questionnaire
TMHI: Thai Mental Health Indicator
